# The effectiveness of teletherapy for developmental language disorder in India

**DOI:** 10.3389/fdgth.2026.1827565

**Published:** 2026-06-23

**Authors:** Irfana Thahani, Juniya Joby, Callum Nicholls, Sangeetha Mahesh, Yasir Ahmed Syed

**Affiliations:** 1 Tele Center for Persons with Communication Disorder (TCPD), All India Institute of Speech and Hearing, Mysore, India; 2 School of Biosciences and Neurosciences and Mental Health Innovation Institute, Cardiff University, Cardiff, United Kingdom

**Keywords:** children, developmental language disorder, India, speech-language pathologists, tele-therapy

## Abstract

**Introduction:**

Developmental language disorder (DLD) is a condition that impairs language acquisition in children despite typical cognitive abilities and the absence of neurological, physical, or hearing impairments. Early intervention through speech therapy is critical, with in-person therapy traditionally considered the standard approach. However, teletherapy delivered via digital platforms has emerged as a potential alternative, particularly in contexts where access to conventional services is limited.

**Methods:**

This study investigated the impact of teletherapy on children aged 2–5 years in India diagnosed with DLD, using a single-group pre–post treatment design. Ten children participated in a structured three-phase process consisting of a pre-therapy assessment, a series of teletherapy sessions, and a post-therapy assessment. The sessions were conducted via video calls and incorporated language stimulation strategies including imitation, modelling, and expansion.

**Results:**

Participants demonstrated improvements in both expressive and receptive language skills following the intervention. Parents reported high levels of satisfaction with the teletherapy approach, highlighting its convenience and accessibility as key advantages. Although some challenges were noted, including technological issues and increased demands on caregivers, these did not substantially impede the observed progress.

**Discussion:**

The findings provide early evidence that teletherapy may be a feasible and accessible approach for supporting language development in children with DLD, particularly in settings with limited access to in-person services. However, given the small sample size and lack of a control group, further research involving larger, controlled studies is necessary to establish the effectiveness of teletherapy and its comparability to traditional therapy models.

## Introduction

Developmental language disorder (DLD) is a developmental delay in expressive language observed in children who have normal nonverbal IQ and no diagnosed neurological, physical, or hearing disorders (1). In India, large-scale studies on the prevalence of DLD are limited. However, smaller studies and clinical observations suggest its prevalence aligns with global estimates of 5%–8% among young children (2). Supporting this, Jijo et al. (3) reported a prevalence rate of 8.04% in India, indicating a significant presence of language delays among children. Unfortunately, awareness among parents remains low, with many unable to recognize early signs of language or speech delays in their children.

Speech therapy plays a crucial role in addressing DLD by offering structured, evidence-based interventions to support language development. Techniques such as modelling, imitation, prompting, and expansion are widely used to foster language learning in interactive, natural settings. These methods not only enhance essential language skills but also improve overall communication and social development, underscoring the critical role of speech therapy in managing DLD.

Traditionally, speech-language pathologists (SLPs) have provided in-person therapy. However, the rise of telehealth, a remote service delivery model has transformed how speech therapy is accessed and delivered (4). Teletherapy enables professionals to conduct assessments and interventions online, providing clients and their families with greater flexibility while eliminating barriers like travel and geographic limitations. In India, the speech-language pathology workforce remains limited relative to the population, with a reported ratio of approximately 4.41 speech-language pathologists per 100,000 population, indicating a significant shortage of service providers (5). In addition to this overall shortage, services are unevenly distributed, with a strong urban concentration that limits access for individuals in rural and remote regions. This results in substantial barriers to timely assessment and intervention for individuals with communication disorders. Studies have consistently highlighted that limited workforce availability, long travel distances, and restricted service coverage contribute to poor accessibility and delayed intervention, particularly in underserved areas (6, 7). Collectively, these constraints emphasize the need for teletherapy as a service delivery model to improve accessibility and extend speech-language pathology services to populations with limited access.

Since the COVID-19 pandemic, telepractice has become increasingly integrated into pediatric speech-language pathology services worldwide. Teletherapy has proven particularly effective for diagnostic assessments and interventions, improving accessibility for individuals who might otherwise struggle to access quality care. Among the conditions treated by SLPs, Developmental Language Disorder is especially critical, as it profoundly affects a child's ability to communicate, influencing academic performance, social relationships, and overall development. Without early, intensive intervention, these challenges often persist into adolescence and adulthood, leading to long-term educational and social setbacks. Evidence-based teletherapy interventions enable children with DLD to improve their communication skills and reach their full potential, empowering them to lead more enriched lives.

Systematic reviews and telepractice studies have reported positive language and communication outcomes associated with teletherapy interventions in children, particularly when caregiver involvement and structured evidence-based strategies are incorporated. Tambyraja et al. (8) examined speech-language teletherapy services delivered during the COVID-19 pandemic in school-aged children in the United States. The study reported that teletherapy improved continuity of care and accessibility for many families, although technological barriers and caregiver burden remained important challenges. Chaudhary et al. (9) explored outcomes of teletherapy in patients with speech-language disorders using a structured questionnaire. The authors conclude telerehabilitation as a reliable method to deliver speech and language services at community level, on a long-term basis as is proven by the high satisfaction scores among clients as well as service providers. Bolden and Grogan-Johnson (10) discussed best practices for paediatric telepractice interventions and highlighted the importance of caregiver involvement, clinician preparation, and interactive online activities in improving engagement during virtual sessions. Kwok et al. (11) explored preschool telepractice implementation and found that flexible service delivery, caregiver participation, and home-based intervention increased accessibility and family satisfaction. Christopoulou et al. (12) reviewed telepractice interventions for children with developmental disorders and reported that caregiver-mediated teletherapy approaches may support communication development and engagement. In a systematic review of digital interventions for children with developmental language disorder. Zhou et al. (13) discovered that technology-based interventions frequently focused on vocabulary, phonological, and grammatical skills, underscoring the expanding role of digital platforms in language intervention. Sikka et al. (14) explored parental perspective on teletherapy sessions for children having speech-language delay and suggested 95% of parents reported improved motivation for speech-language therapy, 90% of them felt it to be cost-effective, but 80% of them believe the need of face-to-face intervention after 6 months. Recent systematic reviews and meta-analyses (15) have also suggested that telepractice interventions may support clinically meaningful speech-language outcomes in pediatric populations when evidence-based approaches are implemented consistently. Further, Jain et al. (16) surveyed 100 parents of children with neurodevelopmental disorders and most parents and therapy providers reported to be beneficial in terms of parents convenience, safety from infection, better parent-child bonding and understanding. Additionally, authors report improvisation in teletherapy services for running smooth sessions.

Teletherapy offers significant benefits, including improved accessibility, reduced travel, time savings, increased privacy, and higher engagement for children, particularly in rural schools. Studies report high satisfaction among parents and school staff, with advantages outweighing drawbacks. However, challenges persist, such as differing views between therapists and clients, technology access issues, and the need for caregiver involvement. Clear communication about caregiver roles is essential to address these barriers effectively. While teletherapy is gaining traction as a tool for speech-language interventions, its impact within the Indian context remains underexplored. This study seeks to address this gap by evaluating the improvements associated with teletherapy in managing Developmental Language Disorder among Indian children. As technology continues to transform healthcare delivery, it is vital to understand how teletherapy can enhance accessibility and improve outcomes for children with language impairments. By delving into this critical area, the research aims to offer valuable insights into the potential of teletherapy, ensuring that children with DLD receive the support they need to thrive in an increasingly digital world.

## Methods

This pilot study utilised a single-group pre-post intervention design. The study was carried out in three distinct phases: Phase 1 involved a pre-therapy assessment, Phase 2 focused on tele-therapy sessions, and Phase 3 concluded with a post-therapy assessment. Quantitative assessment of the improvements observed following the course of therapy was combined with qualitative data collected through interviews, offering insights into participant satisfaction and the challenges encountered during tele-therapy.

### Participants

Ten children aged 2–5 years with clinically diagnosed Developmental Language Disorder (DLD) were recruited from the teletherapy waiting list at the All India Institute of Speech and Hearing (AIISH), where they had been enrolled for services but had not yet started therapy. All were native Malayalam speakers, with occasional exposure to English at home, although Malayalam remained their dominant language. Power analysis using G*Power (asymptotic relative efficiency method) indicated that this gave 80% power to detect an effect size of dz = 1.18 at the 2.5% significance level (following Bonferroni correction). This corresponds approximately to a pseudo median difference of 1.18 × the SD of paired differences.

#### Inclusion criteria

Eligible participants were required to demonstrate normal hearing as well as cognitive, psychological, and neurological abilities appropriate for their age.

#### Exclusion criteria

Participants were excluded if they had any neurological disorders, sensory impairments, or behavioral and emotional conditions that could hinder their ability to participate. Additional developmental or psychiatric diagnoses also rendered individual's ineligible for the study.

### Phase 1: Pre-therapy assessment

To establish a baseline and develop tailored therapy plans, each child's language abilities were assessed using the *Assessment of Speech and Language Skill Checklist* (17), a tool standardized for Indian children. Information was gathered through parental interviews, direct observation, and eliciting responses from the child. This comprehensive checklist evaluates key aspects of language development, including phonology, morphology, syntax, and semantics, providing a detailed profile of the child's speech and language abilities.

Although the ACSLS is standardized and widely used in Indian clinical settings, formal validation studies examining its reliability in tele-assessment contexts are currently limited. To improve consistency during remote assessment, clinicians followed standardized administration procedures and provided caregiver guidance throughout the assessment process.

The checklist is structured into 18 developmental levels, covering ages 0–6 years. Levels for ages 0–3 years are segmented into 3-month intervals, while those for ages 3–6 years are divided into 6-month intervals. It assesses two primary domains: Receptive Language Age (RLA) and Expressive Language Age (ELA).

Additionally, the *Short Sensory Profile-2* (18) was used to evaluate the child's sensory system, behaviors, and sensory processing patterns. A clinical psychologist also conducted a psychological evaluation to assess the child's cognitive functioning, ensuring a holistic understanding of their developmental needs.

### Phase 2: Teletherapy

The second phase focused on delivering therapy sessions through teletherapy, using a reliable video calling platform to ensure consistent virtual interactions between the therapist and the child. Sessions were conducted using internet-based video conferencing platforms such as Google Meet and Zoom, depending on caregiver accessibility and internet availability. Smartphones, tablets, and laptops were used based on family resources and convenience. Parents were thoroughly briefed on the study's objectives, session timings, duration, and the importance of regular attendance. They were also guided on maintaining a stable internet connection and minimizing distractions to create an optimal environment for their child during sessions.

Each child participated in two teletherapy sessions per week, lasting approximately 40 min each, over a span of 12 weeks to ensure steady and measurable progress. The speech therapy sessions incorporated a variety of language stimulation techniques, such as imitation, modeling, prompting, focused stimulation, extension, expansion, and milieu teaching. Therapy goals were carefully set to challenge the child just beyond their current skill level, promoting growth and development. Early sessions emphasized functional communication, equipping the child to use language effectively in real-life situations. Target words were chosen strategically and integrated into the stimulation techniques to reinforce learning. To keep the child engaged, interactive tools like online games, storybooks, PowerPoint presentations, and worksheets were used, making language practice both fun and effective.

### Teletherapy resources utilized

Telepractice incorporated a diverse range of materials, including websites, PDFs, slide presentations, online games, and word-processed documents. A detailed breakdown of the resources can be found in Table 1.

**Table 1:** Tele- therapy resources used in the study.

Resources	Description	Examples
Interactive games and language activities	Interactive materials were accessed through a variety of educational websites. Activities can be chosen that elicit specific speech or language targets or be used as reinforcers.	Boom learning https://wow.boomlearning.com ABCYa https://www.abcya.com/ PBS kids https://pbskids.org/ Pink cat games https://www.pinkcatgames.com
Online stories and books	Stories and books were accessed online through e- book formats, websites offering leveled reading materials, video streaming platforms, and local library resources.	Monkey pen https://monkeypen.com/ Free kids book https://freekidsbooks.org/ Story weaver https://storyweaver.org.in/en/ Story place https://www.storyplace.org/
Video streaming	Video streaming sites were used to engage clients and provide opportunities to elicit and practice language. Short videos can be used to practice summarizing, elicit language samples or augment written materials. Wordless animated video shorts and educational videos can all be used as part of language intervention.	You tube https://youtube.com
Slideshow presentations	Slideshows can be used in edit mode to allow clients to move clip art or therapy stimuli around on the screen. In addition, materials can be viewed in presentation mode and the annotation tools can be utilized.	Customized presentations

### Phase 3: Post-therapy assessment

The final phase focused on evaluating the outcomes of the 12-week Tele-therapy program through a detailed post-therapy assessment. Using the same checklist applied prior to the intervention, a comprehensive reassessment of expressive and receptive language skills was conducted, with particular emphasis on vocabulary and sentence structure. By comparing pre- and post-therapy results, any improvements or changes in the child's language abilities were identified and analyzed.

### Qualitative data collection

To gauge parental satisfaction with the Tele-therapy program, semi-structured online interviews were conducted. Each 45-minute interview, recorded during the post-therapy phase, delved into various aspects such as accessibility, ease of use, and perceived improvements following the therapy. It also explored barriers like technological challenges and facilitators such as convenience and engagement, providing deeper insights into the overall experience. Interview responses were analysed using thematic analysis. The primary researcher reviewed and coded the interview responses to identify recurring themes related to accessibility, satisfaction, engagement, and technological challenges. Themes were further reviewed with the research supervisor to improve consistency and reduce interpretative bias.

### Statistical analysis

Wilcoxon Signed Ranks Tests were conducted using the coin package in R on the paired differences in ELA and RLA levels for each participant. The Hodges–Lehmann pseudo-median difference was used as a test statistic, and the Šidák correction with *N* = 2 was applied to *p*-values and confidence intervals to correct for multiple comparisons. Confidence intervals for the median and interquartile range were computed via bootstrapping with *R* = 2,000 resamples using the boot package in *R*.

## Results

### Participants show a statistically significant increase in language ability following a 12-week teletherapy program

**Figure 1:**
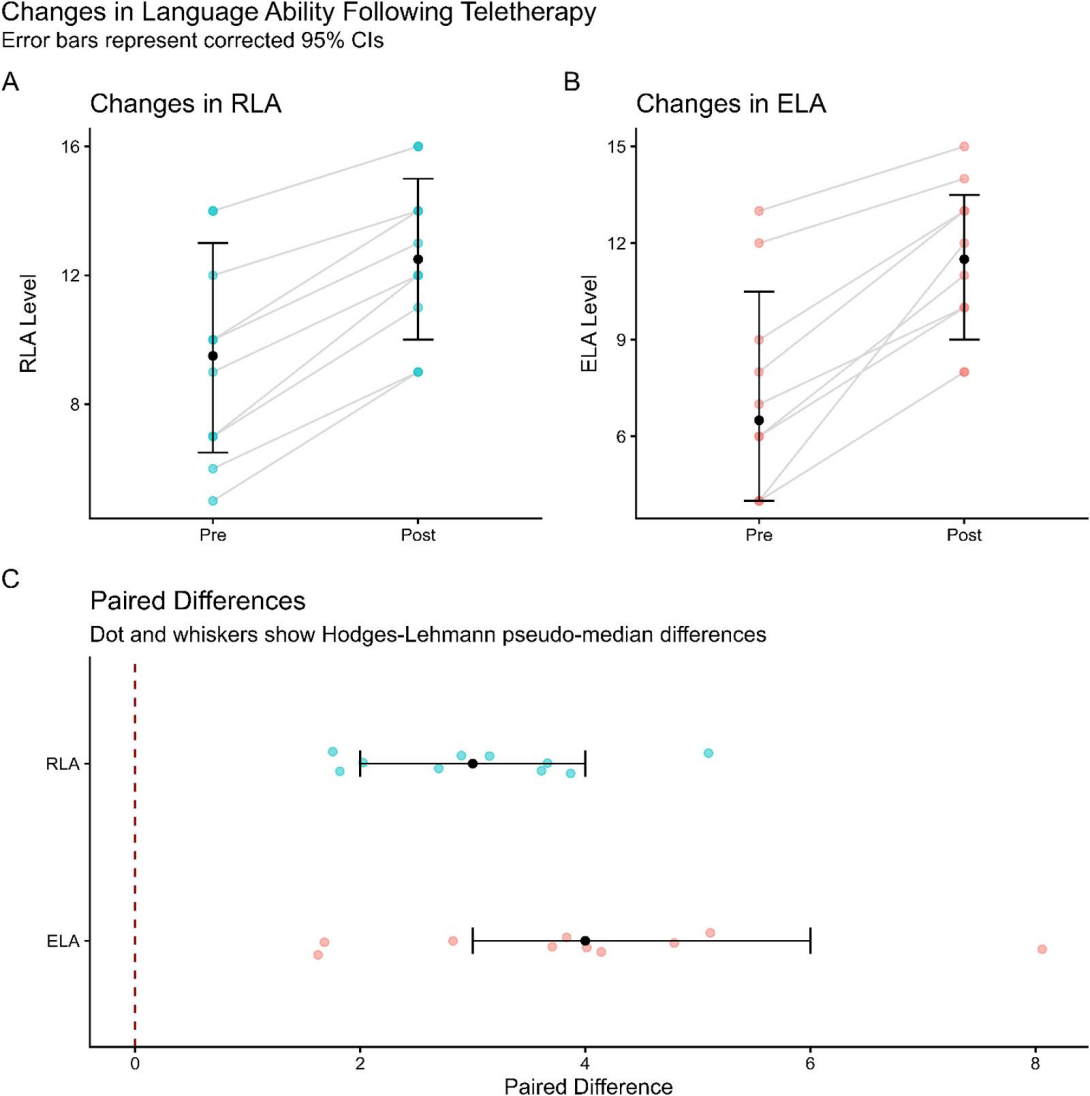
Changes in language ability following teletherapy. **(A)** Changes in receptive language age (RLA) following the teletherapy program. Error bars represent corrected 95% confidence intervals for the median. **(B)** Changes in expressive language age (ELA) following the teletherapy program. Error bars represent corrected 95% confidence intervals for the median. **(C)** Paired differences in RLA and ELA levels following teletherapy. Points represent paired differences between the pre-therapy and post-therapy assessments (offset for visibility). Dot and whiskers represent Hodges–Lehmann pseudo-median differences with corrected 95% CIs.

To assess the efficacy of the 12-week teletherapy program delivered to the participant group, both receptive (RLA) and expressive language age (ELA) levels were measured prior to the commencement and following the completion of the course of 24 sessions. Both RLA and ELA levels increased following the completion of the teletherapy program, with ELA showing the largest increase (Table 1, Figures 1A,B).Variability in both RLA and ELA levels was similar both before and after treatment, though slightly reduced in the post-assessment (Table 1).

The changes in observed language outcomes following teletherapy were assessed for statistical significance using two Wilcoxon signed rank tests, corrected for multiple comparisons. Wilcoxon analysis indicated that both ELA and RLA levels were significantly increased following the course of teletherapy (pseudo-median difference = 4, *V* = 55, *p* = 0.0055 and pseudo-median difference = 3, *V* = 55, *p* = 0.0055 respectively), with the increase in ELA slightly larger (Figure 1C).

On an individual level, participants consistently demonstrated measurable progress in language outcomes, with all individuals achieving at least a two-level increase in both RLA and ELA (Table 2). The results obtained here indicate substantial and rapid language development within a relatively short timeframe, suggesting notable improvements following the teletherapy intervention.

**Table 2:** Summary statistics for changes in language outcomes.

Language outcome	Pre-assessment	Post-assessment	Differences
RLA	Median: 9.5 [6.5, 13]	Median: 12.5 [10, 15]	Median: 3 [2, 4]
	IQR: 2.5 [1, 7.75]	IQR: 1.5 [0.75, 6.5]	IQR: 1 [0, 2.5]
ELA	Median: 6.5 [4, 10.5]	Median: 11.5 [9, 13.5]	Median: 4 [2.5, 5]
	IQR: 2.5 [1, 8.25]	IQR: 1.5 [0.75, 6]	IQR: 1 [0, 4.75]

Values shown are the median and interquartile range, with bootstrapped, Šidák-corrected 95% confidence intervals.

### Core themes from parental feedback on teletherapy

A qualitative analysis of parental feedback provides valuable context to quantitative findings, shedding light on both the benefits and challenges of teletherapy. Four core themes emerged:

1. Accessibility and convenience

Parents overwhelmingly praised teletherapy for its convenience, highlighting the elimination of commute times and the flexibility of scheduling sessions. These factors made it far easier to incorporate therapy into their busy routines.

2. Perceived effectiveness

Many parents reported noticeable improvements in their children's communication abilities, citing enhanced vocabulary, improved sentence structure, and greater confidence in self-expression. These observations underscore teletherapy's effectiveness in fostering meaningful progress.

3. Engagement through interactive tools

Interactive tools and multimodal activities emerged as standout features of teletherapy. Storytelling, games, and visual aids were particularly effective in capturing children's attention, keeping them motivated, and creating an engaging and enjoyable learning environment.

4. Overcoming technological challenges

While some families initially faced technical hurdles—such as internet connectivity issues, device incompatibility, or software glitches—these challenges were largely temporary. Parents with limited technical skills found the learning curve manageable with guidance from therapists. As families adapted, sessions became smooth and productive.

This analysis highlights teletherapy's potential to deliver accessible, effective, and engaging care despite occasional technological setbacks.

By combining quantitative data with qualitative insights, the study supports teletherapy as an effective and accessible mode of service delivery alongside traditional in-person therapy. It not only delivers measurable improvements in language development but also offers a flexible, user-friendly experience. Parents who actively participated in therapy sessions reported greater satisfaction, emphasizing the importance of consistent engagement in achieving positive outcomes.

Ultimately, this study showcases the transformative power of teletherapy—not just in boosting language skills, but in empowering families through accessibility, flexibility, and innovative approaches to learning.

## Discussion

This study employed a single-group pre-post design to evaluate teletherapy for children with Developmental Language Disorder (DLD) and demonstrated significant improvements in both receptive and expressive language skills over a 12-week intervention period. These results provide preliminary evidence for the feasibility and efficacy of teletherapy, particularly in cases where access to traditional therapy is limited by geographical or practical constraints. Pre-therapy assessments using the Assessment of Speech and Language Skills Checklist (17) showed delays in language skills, with children performing below their age level. Post-therapy assessments revealed notable progress in both receptive and expressive language abilities, helping children better communicate and understand language in daily life. These findings highlight the value of evidence-based teletherapy interventions in bridging service gaps for children with DLD.

Participants demonstrated impressive progress in both receptive and expressive language skills, with all achieving at least a two-level improvement on the ACSLS scale. Individual pre- and post-therapy assessment outcomes for all participants are presented in Table 3. Significant gains were observed in areas such as vocabulary comprehension, sentence construction, following directions, and overall communication abilities. Prior to therapy, many participants struggled to form even basic sentences. By the end of the sessions, they were confidently constructing longer, more complex sentences with ease. This remarkable progress was the result of targeted speech-language therapy, which emphasized key areas like grammatical structures (e.g., conjunctions and tense agreement), sentence expansion, and modelling techniques to enhance expressive language skills. The teletherapy sessions incorporated engaging tools such as interactive online stories, educational games, and PowerPoint presentations to create a dynamic and improved learning environment. These resources captivated the children's attention, facilitating the acquisition of new vocabulary and improving their ability to follow instructions. By leveraging multimodal materials, the therapy allowed children to interact with language in varied and meaningful ways, fostering both comprehension and the ability to tackle diverse linguistic tasks. The observed improvements can be attributed to evidence-based strategies employed during teletherapy, including modelling, imitation, and sentence expansion (23) all of which have been previously shown to support language development in children with language impairments in in-person (19) and virtual settings (16). Through consistent modeling and guided verbal practice, children expanded their vocabulary and developed the ability to construct more sophisticated sentences. This targeted approach led to significant advancements in their communication skills, marking a meaningful step forward in their language development journey.

**Table 3:** Pre and post-therapy assessment results.

Sl.No	Age/Sex	Pre-therapy assessment (ACSLS)	Post-therapy assessment (ACSLS)
1	3 years/M	RLA:1.1–1.3 years (Level 5)	RLA: 2.1–2.3 years (Level 9)
		ELA:0.10–1.0 years (Level 4)	ELA: 1.10–2.0 years (Level 8)
2	2.11 years/M	RLA:1.4–1.6 years (Level 6)	RLA: 2.1–2.3 years (Level 9)
		ELA: 0.10–1.0 years (Level 4)	ELA: 1.10–2.0 years (Level 8)
3	3 years/M	RLA:1.7–1.9 years (Level 7)	RLA: 2.10–3.0 years (Level 12)
		ELA:0.10–1.0 years (Level 4)	ELA: 2.10–3.0 years (Level 12)
4	2.11 years/M	RLA:1.7–1.9 years (Level 7)	RLA: 2.7–2.9 years (Level 11)
		ELA:1.4–1.6 years (Level 6)	ELA: 2.7–2.9 years (Level 11)
5	3.5 years/F	RLA:2.1–2.3 years (Level 9)	RLA:2.10–3.0 years (Level 12)
		ELA:1.4–1.6 years (Level 6)	ELA:2.4–2.6 years (Level 10)
6	4.4 years/M	RLA:2.4–2.6 years (Level 10)	RLA: 3.7–4.0 years (Level 14)
		ELA:1.10–2.0 years (Level 8)	ELA: 3.0–3.6 years (Level 13)
7	3.4 years/M	RLA:2.4–2.6 years (Level 10)	RLA: 3.0–3.6 years (Level 13)
		ELA:1.7–1.9 years (Level 7)	ELA: 2.4–2.6 years (Level 10)
8	4.5 years/M	RLA:2.10–3.0 years (Level 12)	RLA: 3.7–4.0 years (Level 14)
		ELA:2.1–2.3 years (Level 9)	ELA: 3.0–3.6 years (Level 13)
9	5 years/F	RLA: 3.7–4.0 years (Level 14)	RLA: 4.7–5.0 years (Level 16)
		ELA: 2.10–3.0 years (Level 12)	ELA: 3.7–4.0 years (Level 14)
10	5 years/M	RLA: 3.7–4.0 years (Level 14)	RLA: 4.7–5.0 years (Level 16)
		ELA: 3.0–3.6 years (Level 13)	ELA: 4.1–4.6 years (Level 15)

The language techniques used in this intervention significantly supported the child's language development, leading to notable improvements in communication and social engagement. By combining evidence-based strategies, the child progressed from producing short, simple utterances to forming longer, grammatically correct sentences. These techniques created a structured environment for practicing language skills in meaningful contexts, enhancing both comprehension and expression. Visual supports improved understanding, while structured communication approaches provided a clear framework for social interaction. Over time, the child became more independent in communication, relying less on prompts and using language naturally. This holistic approach fostered not only language development but also confidence in functional communication across various contexts.

Parental involvement is a cornerstone of successful teletherapy programs, playing a pivotal role in ensuring therapy techniques are consistently reinforced and practiced at home. Parents reported that teletherapy offered notable advantages, including greater convenience, accessibility, and flexibility, particularly due to the elimination of travel time and the ability to conduct sessions from home. They also found it easier to integrate therapy activities into daily routines, further enhancing the program's effectiveness. While these benefits were appreciated, challenges like network issues and device navigation occasionally disrupted sessions. Despite this, parents expressed overwhelmingly positive feedback, noting that the consistent support of teletherapy outweighed minor technical setbacks.

Research globally has consistently shown teletherapy is as effective as direct or hybrid service delivery (20, 21, 24). Findings align with earlier research, such as Sikka (14), which emphasized the importance of parental involvement and teletherapy's flexibility in achieving successful language intervention. Caregiver involvement was crucial for maintaining the child's engagement and applying therapeutic strategies outside of sessions. These findings align with those of Tambyraja et al. (8), who also noted challenges in rural teletherapy programs. These are also consistent with earlier Indian telepractice studies that reported improved accessibility and feasibility of teletherapy services for individuals residing in rural and underserved regions (6, 7). Overall, the study highlights teletherapy as an effective, accessible solution for managing language impairments in children while acknowledging areas for improvement in technology and connectivity. Similarly, Jain et al. (16) iterated benefits with telehealth program but also emphasize on improvisations in implementation and delivery system of tele therapy services are needed for running smooth sessions.

In the Indian context, this study provides valuable insights into the viability of teletherapy as an intervention for children with language impairments. While telepractice research is well-established in western countries, it is still in its nascent stages in India. By combining evidence from prior research with the outcomes of this study, it becomes clear that teletherapy, particularly when integrated with hybrid approaches, offers a flexible and impactful intervention method. This is especially relevant in contexts where access to in-person therapy is limited. These findings underscore the promise of teletherapy in bridging service delivery gaps in speech-language pathology, paving the way for more inclusive and accessible care. While teletherapy has demonstrated its effectiveness in various international contexts, this study is among the first, to our knowledge, to explore its impact on children with DLD in India. The promising outcomes of this intervention suggest that teletherapy could play a vital role in addressing the service delivery gap for children who may otherwise lack access to traditional therapy. However, further research is needed to examine its long-term effects on language development and to address challenges related to technology and caregiver involvement.

In conclusion, this research provides preliminary evidence supporting the effectiveness of teletherapy for children with Developmental Language Disorder (DLD). Significant gains were observed in both receptive and expressive language skills, with children showing notable progress in vocabulary development, sentence structure, and comprehension. The success of teletherapy was largely attributed to its flexibility, accessibility, and the active involvement of parents. Although some technological challenges were encountered, the findings suggest that teletherapy is a viable option, particularly in areas where in-person services are not readily available. Future research should focus on refining teletherapy techniques, addressing technological limitations, and exploring its potential for treating other speech and language disorders.

### Limitations

The present study constitutes a successful and compelling pilot suggesting that further, larger scale investigations of teletherapy methods in speech and language therapy are warranted. However, while participants in this study demonstrated a statistically significant improvement in both expressive and receptive language age, it is important to keep in mind that this study used a small sample size of 10 participants. Subsequent replications will utilise a large sample size to validate the findings described here.

Furthermore, this study utilised a single-group pre-post design without a control or comparison group. Therefore, improvements observed over the 12-week period cannot be conclusively attributed to teletherapy alone, as developmental maturation, increased parental involvement, repeated language exposure, or environmental influences may also have contributed to the observed gains. It is also important to note that there is a potential for response bias in the parental report questionnaires. In addition, although the ACSLS is standardized for Indian children, its psychometric properties in tele-assessment settings have not yet been extensively validated.

One unaddressed aspect of the teletherapy intervention detailed in this study is the retention of the observed improvements in language skills. Therefore, subsequent studies should include an additional follow-up assessment of participants to verify that language skills remain elevated over control individuals.

## Data Availability

The datasets presented in this article are not readily available because. Requests to access the datasets should be directed to irfavanafavaz123@gmail.com.
